# Biosynthesis of polyhydroxyalkanoates containing hydroxyl group from glycolate in *Escherichia coli*

**DOI:** 10.1186/s13568-016-0200-5

**Published:** 2016-04-14

**Authors:** Chayatip Insomphun, Shingo Kobayashi, Tetsuya Fujiki, Keiji Numata

**Affiliations:** Enzyme Research Team, RIKEN Center for Sustainable Resource Science, 2-1 Hirosawa, Wako-shi, Saitama, 351-0198 Japan; Kaneka Corporation, 1-8 Miyamae-cho, Takasago-cho, Takasago, Hyogo 676-8688 Japan

**Keywords:** Polyhydroxyalkanoates, Hydroxyl group, *Escherichia coli*, Glycolate

## Abstract

Polyhydroxyalkanoates (PHAs) containing hydroxyl groups in a side chain were produced in recombinant *Escherichia coli* JM109 using glycolate as the sole carbon source. The propionate-CoA transferase (*pct*) gene from *Megasphaera elsdenii* and the β-ketothiolase (*bktB*) gene and *phaCAB* operon from *Ralstonia eutropha* H16 were introduced into *E. coli* JM109. A novel monomer containing a hydroxyl group, dihydroxybutyrate (DHBA), was the expected product of the condensation of glycolyl-CoA and acetyl-CoA by BktB. The recombinant strain produced a PHA containing 1 mol% DHBA. The incorporation of DHBA may have been restricted because the expression of *phaAB1* competes for acetyl-CoA. The PHA containing DHBA units were evaluated regarding thermal properties, such as melting temperature, glass transition temperature and thermal degradation temperature. The current study demonstrates a potential use of PHA containing hydroxyl groups as renewable resources in biological materials.

## Introduction

Polyhydroxyalkanoates (PHAs), which are one of biodegradable polyesters, are produced intracellularly by a wide variety of microorganisms from carbon and energy storage compounds under unbalanced growth conditions (Doi et al. 1995; Madison and Huisman 1999; Rehm 2007). Because of their biodegradability, biocompatibility, and thermoprocessability, PHAs have attracted much attention for applications in agriculture, medicine, packaging and the food industry (Chen 2009; Numata et al. 2009; Sudesh and Iwata 2008; Verlinden et al. 2007). Poly(3-hydroxybutyrate), P(3HB), is the most abundant PHA in nature. However, the application of P(3HB) is limited by its high crystallinity and brittle nature (Sudesh et al. 2000). A variety of PHAs containing 3-hydroxybutyrate (3HB) and a second monomer, such as 3-hydroxypropionate (3HP), 4-hydroxybutyrate (4HB), 5-hydroxyvalerate (5HV), and 3-hydroxyhexanoate (3HHx), have been produced to improve material properties (Budde et al. 2011; Chuah et al. 2013; Fukui et al. 2009; Saito et al. 1996). The mechanical and thermal properties of PHA relate to its monomer composition, which depends on the type of carbon source used, the available metabolic pathway for PHA biosynthesis, and the substrate specificity of PHA synthase (PhaC) and related enzymes (Lu et al. 2009; Numata et al. 2012; Steinbüchel and Valentin 1995; Yamashita et al. 2006). Among various PHAs, those containing functional groups, such as benzyl, hydroxyl and carboxyl groups, are more attractive because the functional group side chain can improve their physical properties (Hazer and Steinbüchel 2007). Moreover, some reactive functional groups in PHA may be further modified by chemicals to extend their applications.

**Fig. 1:**
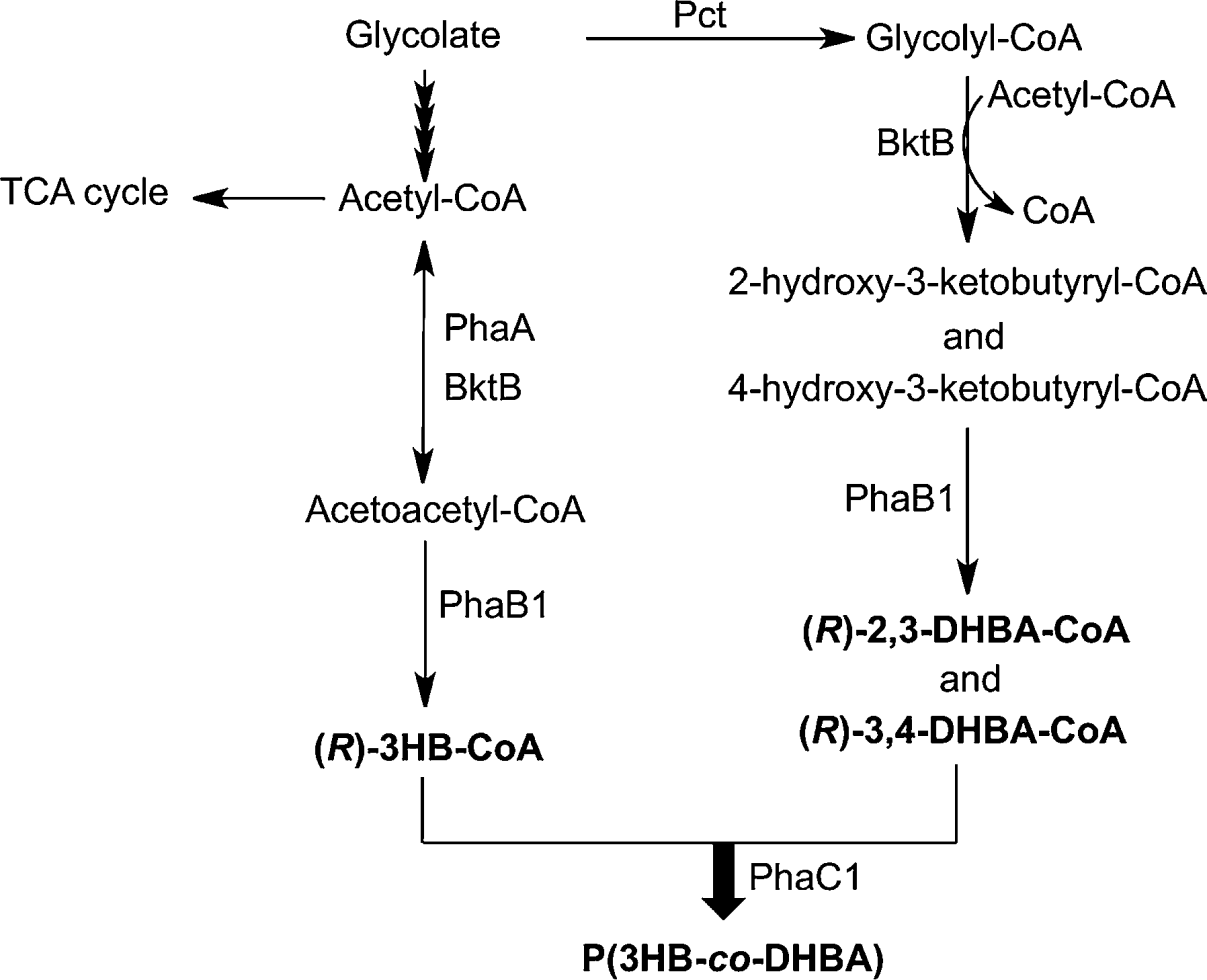
Proposed biosynthesis pathway for the production of PHA containing a DHBA monomer in *E. coli*

PHA with pendant hydroxyl groups has been prepared by chemical modification of unsaturated PHAs using potassium permanganate or a borane-tetrahydrofuran complex (Lee et al. 2000; Renard et al. 2005). However, it is not easy to control the optical purity of these products. Recently, Martin et al. have constructed a metabolic pathway of dihydroxybutyrate (DHBA) biosynthesis in *Escherichia coli* by the introduction of the propionate-coenzymeA transferase gene (*pct*) from *Megasphaera elsdenii*, the β-ketothiolase gene (*bktB*) and 3-hydroxybutyryl-coenzymeA dehydrogenase gene (*phaB*) from *Ralstonia eutropha* H16, and the thioesterase (*tesB*) gene from *E. coli* MG1655 (Martin et al. 2013). Their results have indicated the possibility of incorporating a DHBA unit into PHA by using the *pct*, *bktB* and *phaB* genes. We proposed a pathway for the production of PHA containing a DHBA monomer, as shown in Fig. 1. Therefore, in this study, we demonstrated the biosynthesis of PHA containing side chains with hydroxyl groups in *E. coli* JM109 by introducing *pct* from *M.elsdenii* and the *bktB* and *phaCAB* operons from *R. eutropha* H16. Furthermore, the thermal properties and stabilities of the PHA with hydroxyl groups were characterized to determine the effects of the side chains.

## Materials and methods

### Bacterial strains and culture conditions

The bacterial strains and plasmids used in this study are listed in Table 1. *Escherichia coli* strains were grown at 37 or 30 °C on a Luria–Bertani (LB) medium (Becton, Dickinson and Company, NJ, USA) containing tryptone (10 g/L), yeast extract (5 g/L) and NaCl (5 g/L), and 100 µg/ml kanamycin and 100 µg/ml ampicillin were added to the medium when necessary.

**Table 1:** Bacterial strains and plasmids used in this study

Strain or plasmid	Relevant marker	Source or reference
*Escherichia coli*		
JM109	F′ *traD36 proA* ^+^ *B* ^+^ *lacI* ^*q*^Δ*(lacZ)M15/*Δ*(lac*-*proAB) glnV44 e14* ^−^ *gyrA96 recA1 relA1 endA1 thi hsdR17*	Invitrogen
Plasmids		
pBBR-CAB	pBBR1-MCS2 derivative, *phaCAB*	(Ushimaru et al. 2014)
pTrcHis2B	pBR322 *ori*, *P*_*trc*_, *lacI* ^*q*^, Amp^r^	Invitrogen
pTrc-pct	pTrcHis2B derivative, *pct*	This study
pTrc-pct-bktB	pTrcHis2B derivative, *pct*, *bktB*	This study

### Construction of plasmids and strains

DNA manipulations were conducted according to standard procedures (Sambrook and Russell 2001). The sequences of the oligonucleotide primers used in this study are listed in Table 2. A synthetic *pct* gene from *Megasphaera elsdenii* (DDBJ accession number: LC126829, 1554 bp) with codon optimization was synthesized by Operon Biotechnologies (Tokyo, Japan). The ribosome binding site and linker (AAAGGAGGAACAACC) were added, and the synthesized fragment was digested by *Eco*RI and *Bam*HI and then inserted into the pBBR1-MCS2 vector at the corresponding sites to obtain the pBBR-pct plasmid.

**Table 2:** Sequences of the primers used in this study

Primer	Sequence (5′–3′)	Restriction site^a^
bktB-Fw*Eco*RI	CCGGAATTCAAGGAGGAATAAATGACGCGTGAAGTGGTAGTGG	*Eco*RI
bktB-Rv*Xba*I	TGCTCTAGAGCTCAGATACGCTCGAAGATGGCG	*Xba*I
pct-Fw*Bam*HI	CGCGGATCCAATGCGCAAGGTGGAGATTATC	*Bam*HI
pct-Rv*Eco*RI	CCGGAATTCTCACTTCTTCAGGCCCATCGG	*Eco*RI

^a^Indicated by underlining in the primer sequence

The pTrc-pct plasmid was constructed as follows. The *pct* gene was amplified with pBBR-pct and pct-Fw*Bam*HI/pct-Rv*Eco*RI as the template and primer, respectively. The amplified fragment was digested by *Bam*HI and *Eco*RI and then inserted into the pTrcHis2B vector at the corresponding sites to obtain the pTrc-pct plasmid. A coding region of *bktB* (H16_A1446, 1540 bp) was amplified by PCR with genomic DNA from *R. eutropha* H16 and bktB-Fw*Eco*RI/bktB-Rv*Xba*I as the template and primer, respectively. The amplified fragment was digested by *Eco*RI and *Xba*I and then inserted into the pTrc-pct vector at the corresponding sites to obtain the pTrc-pct-bktB plasmid.

pBBR-CAB was constructed as previously described (Ushimaru et al. 2014).

### Cultivation conditions

PHA production by recombinant *E. coli* was conducted in LB medium at 37 °C until an OD_600_ was reached 0.6. Then, PHA production was induced by adding a 1.0 % (w/v) filter-sterilized glucose solution or different concentrations of filter-sterilized solutions of sodium glycolate [0.25, 0.50, 0.75, and 1.0 % (w/v)] and isopropyl β-D-1-thiogalactopyranoside (IPTG) (final concentration of 1 mM), and the bacteria were cultured at 30 °C for 72 h. Then, the cells were harvested, washed three times with distilled water, and lyophilized.

### Enzyme assay

Acetoacetyl-CoA, propionyl-CoA, and citrate synthase (≥100 units/mg protein) were purchased from Sigma Aldrich (Saint Louis, MO, USA). Recombinant *E. coli* was cultivated in LB medium at 37 °C until an OD_600_ was reached. IPTG was added to a final concentration of 1 mM, and cells continued to grow at 30 °C overnight. The cells were collected by centrifugation at 7500 rpm for 10 min, washed with 20 mM Tris–HCl buffer, pH 7.4, and resuspended in the same buffer (1 g of wet cell/5 ml buffer). The cells were disrupted by sonication (amplitude = 25, process time = 5 min, pulse on = 10 s, and pulse off = 20 s), and cell debris was removed by centrifugation (12,000 rpm, 4 °C, 10 min) to obtain cell free extract. The activity of Pct was determined in the cell free extract by a spectroscopic assay (Lindenkamp et al. 2013). The 400 μl reaction mixture contained 100 mM Tris–HCl buffer, pH 7.4; 200 mM sodium acetate; 20 mM oxaloacetate; 2 mM propionyl-CoA; 1 mM 5,5′-dithiobis-(2-nitrobenzoic acid) (DTNB); 8 units of citrate synthase; and cell free extract. Absorbance was measured at 412 nm. One unit was defined as the activity necessary to produce 1 μmol of thionitrobenzoic acid (TNB) corresponding to the released free CoA-SH per minute (ε_412_ = 14,140 M^−1^ cm^−1^). β-ketothiolase was assayed in a 400 μl reaction mixture containing 50 mM Tris–HCl buffer, pH 7.4; 0.1 mM acetoacetyl-CoA; 1 mM coenzyme A trilithium salt; 25 mM MgSO_4_; and cell free extract. The decreased absorbance was measured at 304 nm (Mifune et al. 2010). One unit was defined as the activity necessary to produce 1 μmol of Mg^2+^-3-ketoacyl-CoA complex per minute (ε_304_ = 19.5 × 10^3^ M^−1^ cm^−1^).

### Polymer extraction from cells

The polymer was extracted from the lyophilized cells by stirring the cells in chloroform for 3 days at room temperature. Cell debris was removed by filtration, and the polymer solution was purified by precipitation with tenfold volumes of hexane and methanol (Mifune et al. 2008). The purified polymer was vacuum dried before weighing.

### Polymer analyses

PHA content within the cells was determined by gas chromatography-mass spectrometry (GC/MS) after ethanolysis of the dried cells with ethanol-chloroform-hydrochloric acid at 100 °C for 4 h, as described previously (Findlay and White 1983). Polymer composition was determined by ^1^H nuclear magnetic resonance (NMR) spectra (VARIAN NMR System 500, Varian, Palo Alto, CA). The 500 MHz ^1^H NMR spectra were recorded in a CDCl_3_ solution of PHA (20 mg/ml) at 27 °C.

The molecular weights of the polymers were obtained using gel-permeation chromatography (GPC) and a viscometer (Numata et al. 2005). GPC measurements were performed at 40 °C using a JASCO GPC system (RI2031, PU-2086, AS-202055, CO2056; JASCO, Tokyo, Japan) with a Shodex K-806 M, K802, and K-G column at 40 °C. Chloroform was used as the mobile phase at a flow rate of 0.8 ml/min, and the sample concentration used was approximately 1 mg/ml. The calibration curve used for estimating the molecular weight was generated using polystyrene standards with low polydispersity. The molecular weights of the nine polystyrene standards were 3148843, 1074876, 460595, 156528, 66001, 28517, 10112, 3252, and 1319 g/mol.

To determine the molecular weight by viscometry, viscosity was measured at 30 °C using a capillary glass Ubbelohde (SU 026130-0003, SIBATA) with a thermostat water bath (VB-3T, SIBATA, Saitama, Japan). For measurement, 13 ml of solution was transferred into the Ubbelohde and incubated in the thermostat water bath for 15 min. The flow time of the PHA solution was determined as the mean of three measurements. To obtain intrinsic viscosity, four PHA concentrations were measured. Sample concentrations varied within 100–175 mg of PHA per 100 ml of chloroform. Molecular weights were calculated from the Mark-Houwink-Coon equation.

Differential scanning calorimetry (DSC) data were recorded at temperatures ranging from −50 to 200 °C on a Perkin-Elmer DSC 8500 instrument (Perkin-Elmer, Waltham, MA) equipped with a cooling accessory under a nitrogen flow rate of 20 ml/min. The melt-crystallized films (approximately 3 mg) were encapsulated in aluminum pans and heated to 200 °C at 20 °C/min, according to a previous report (Numata et al. 2004). The melting temperature (*T*_*m*_) and enthalpy of fusion (Δ*H*_*m*_) were determined from the DSC endotherms. The glass transition temperature (*T*_*m*_), which was analyzed based on the DSC curves from the second heating, was taken as the midpoint of the heat capacity change.

The thermal degradation temperature of PHA was determined by thermogravimetric analysis (TGA) using a TGA/DSC2 STAR^e^ system (Mettler Toledo, Switzerland). PHA (approximately 3 mg) was encapsulated in aluminum pans and heated from 30 to 400 °C at a heating rate of 20 °C/min under a nitrogen atmosphere.

## Results

### Construction of the recombinant strain

The PHA biosynthesis pathway in *E. coli* JM109 was constructed by introducing the plasmid containing the *phaCAB* operon from *R. eutropha* H16. DHBA units were generated by co-expressing the propionate-coenzyme A transferase gene (*pct*) from *M. elsdenii* and the β-ketothiolase gene (*bktB*) from *R. eutropha* H16. The first step of this pathway is the formation of glycolyl-CoA from glycolate by Pct. Glycolyl-CoA is condensed with acetyl-CoA to form 2-Hydroxy-3-ketobutyryl-CoA and 4-Hydroxy-3-ketobutyryl-CoA by BktB (Fig. 1). The two intermediates are reduced to 3,4-dihydroxybutyryl-CoA (3,4-DHBA) and 2,3-dihydroxybutyryl-CoA (2,3-DHBA), respectively, by 3-hydroxybutyryl-CoA dehydrogenase (PhaB1). Finally, 3,4-DHBA and 2,3-DHBA are polymerized with 3-hydroxybutyryl-CoA (3HB-CoA) by PHA synthase1 (PhaC1).

The enzymatic activity of Pct and β-ketothiolase in the recombinant *E. coli* JM109 was determined by spectroscopic assay to confirm gene expression. Our results showed that the cell-free extract from recombinant *E. coli* JM109 harboring pBBR-CAB and pTrc-pct-bktB exhibited both Pct and β-ketothiolase activity. The specific activities of Pct and β-ketothiolase were 7.2 and 4.2 U/mg, respectively. The activity of the blank, containing no Pct or β-ketothiolase, was subtracted from each sample as background. These results confirmed that the *pct* and *bktB* genes were successfully expressed in *E. coli* JM109.

### Biosynthesis of PHA in recombinant *E. coli* JM109

Recombinant *E. coli* JM109 was cultured in LB medium supplemented with 1 % (w/v) glucose or various concentrations of glycolate. The PHA contents were determined by GC/MS (Table 3), and the PHA content of the recombinant strain grown in glucose was approximately 6 wt%. However, the PHA contents of the recombinant *E. coli* JM109 were relatively low (<1 wt%) for all glycolate concentrations used. Cell growth and PHA content decreased when the cells were grown with 0.75 and 1.0 % (w/v) glycolate. This result indicated that high concentrations of glycolate had an inhibitory effect on cell growth and PHA production. Therefore, 0.5 % (w/v) glycolate was selected for further polymer production.

**Table 3:** Biosynthesis of PHA in recombinant *E. coli* JM109 using glucose and glycolate as the sole carbon sources

Carbon sources [%(w/v)]	CDW (g/L)	PHA content (wt%)
1.0 % glucose	0.67 ± 0.03	5.5 ± 0.5
0.25 % glycolate	0.31 ± 0.10	0.9 ± 0.3
0.50 % glycolate	0.47 ± 0.01	0.9 ± 0.2
0.75 % glycolate	0.42 ± 0.02	0.7 ± 0.1
1.0 % glycolate	0.39 ± 0.01	0.7 ± 0.03

### Characterization of PHA

**Fig. 2:**
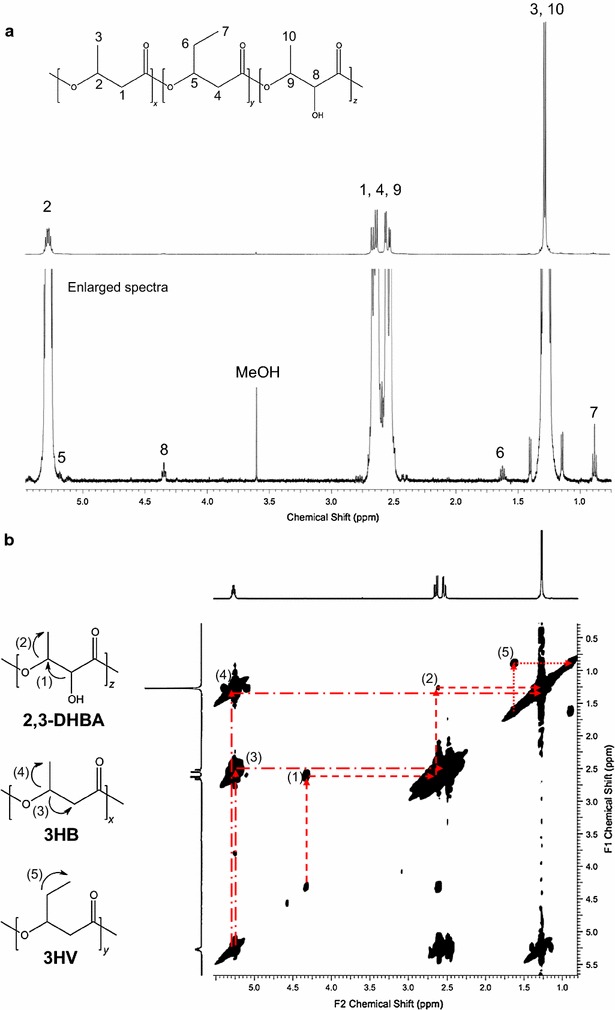
NMR analysis of P(3HB-*co*-1 mol% 3HV-*co*-1 mol% 3,4-DHBA) produced in *E. coli* JM109 harboring pBBR-CAB and pTrc-pct-bktB. **a** 500 MHz ^1^H NMR spectra and **b** ^1^H-^1^H COSY spectra

The composition of the extracted polymer was investigated with ^1^H NMR and 2D (^1^H–^1^H) COSY NMR spectroscopy, which revealed that in addition to 3HB units, 3HV units and novel monomers containing side chains with hydroxyl groups and 2,3-DHBA were incorporated into PHA. The mole fraction of the 3HV and 2,3-DHBA monomers was 1 mol%. The completed assignments of the ^1^H-NMR spectra in CDCl_3_ are shown in Fig. 2a. The methyl protons of 3HV were assigned at δ0.7–0.8 ppm. The methyl protons of 2,3-DHBA and 3HB were assigned at the regions of δ1.1–1.4 ppm. The methylene protons of 3HV were assigned at δ1.7–1.8 ppm. The methylene protons of 2,3-DHBA and 3HB were assigned at δ2.4–2.8 ppm. The oxymethine proton connected to the hydroxyl group was assigned at the region of δ4.3–4.4 ppm. The oxymethine protons of 3HB and 3HV were around δ5.2–5.9 ppm.

Figure 2b shows the COSY spectrum. The signal (1) between the methine proton connected to the hydroxyl group of 2,3-DHBA and the oxymethine proton of 2,3-DHBA appeared at δ4.3/2.6 ppm. The coupling (2) between the oxymethine proton of 2,3-DHBA and the methyl protons of 2,3-DHBA was seen at δ2.6/1.3 ppm. The coupling (3) between the oxymethine proton of 3HB and the methylene protons of 3HB was assigned at δ5.3/2.6 ppm, whereas the coupling (4) between the oxymethine proton of 3HB and the methyl protons of 3HB appeared at δ5.3/1.3 ppm. The coupling (5) between the methylene protons of 3HV and the methyl protons of 3HV was seen at δ1.6/0.9 ppm.

It has been reported that the molecular weight of PHA can be affected by PHA synthase activity, the type and concentration of carbon source (Sim et al. 1997; Taidi et al. 1994). The recombinant *E. coli* JM109 produced a polymer with a number-average molecular weight (*M*_*n*_) of 0.5 × 10^6^ g/mol and polydispersity index (PDI) of 3.2 from glycolate.

### Thermal analysis

Incorporation of 3HV and 2,3-DHBA monomers into P(3HB) had an effect on its thermal properties (Table 4). The *T*_*m*_, Δ*H*_*m*_, and *T*_*g*_ values were lower than those of the P(3HB) homopolymer. However, the Δ*H*_*m*_ value was higher than that of P(3HB-*co*-2 mol% 3HV). The increase in Δ*H*_*m*_ may be due to the incorporation of 2,3-DHBA and its effects on the nucleation of crystallinity.

**Table 4:** Thermal properties of P(3HB), P(3HB-*co*-3HV) and P(3HB-*co*-DHBA)

Samples	*T*_*g*_ (°C)	*T*_*m*_ (°C)	Δ*H*_*m*_ (Jg^−1^)
P(3HB) (Shimamura et al. 1994)	4.0	177.0	97.0
P(3HB-*co*-2 mol% 3HV) (Lee et al. 2008)	−0.7	168.0	65.2
P(3HB-*co*-1 mol% 3HV-*co*-1 mol%-2,3-DHBA)	0.1	168.8	90.3

**Fig. 3:**
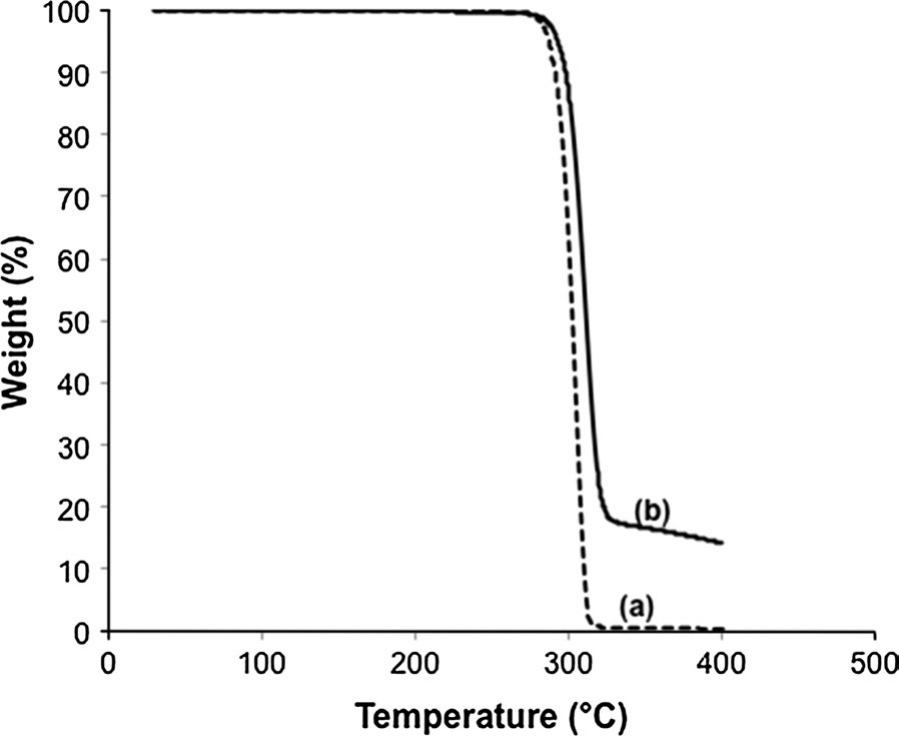
TGA curve of PHA samples. **a** P(3HB) and **b** P(3HB-*co*-1 mol% 3HV-*co*-1 mol% 3,4-DHBA)

To investigate the thermal degradation behavior of P(3HB-*co*-1 mol% 3HV-*co*-1 mol%-2,3-DHBA), TGA was used. Figure 3 shows the TGA curves with a heating rate of 20 °C/min. The TGA curves decreased with temperature due to the thermal degradation of PHA, and there was no char left from P(3HB). The thermal degradation temperatures of 1, 5 and 10 wt% weight loss (*T*_1 %_, *T*_5 %_ and *T*_10 %_) for P(3HB) and P(3HB-*co*-1 mol% 3HV-*co*-1 mol%-2,3-DHBA) are listed in Table 5. The thermal decomposition for P(3HB-*co*-1 mol% 3HV-*co*-1 mol%-2,3-DHBA) at 1, 5, and 10 % weight loss was higher than that for P(3HB).

**Table 5:** Thermal degradation temperatures of PHA samples

Samples	*T*_1 %_ (°C)	*T*_5 %_ (°C)	*T*_10 %_ (°C)
P(3HB)	277.7	287.0	290.9
P(3HB-*co*-1 mol% 3HV-*co*-1 mol%-2,3-DHBA)	280.7	293.0	297.7

## Discussion

PHAs have attracted attention as an alternative to petroleum-based plastic because of their reproducibility from renewable resources. PHAs also show potential in a wide range of applications due to their biodegradability, biocompatibility, and thermoprocessability. To extend their properties and applications, PHAs containing functional groups have been synthesized using several types of microorganisms. *Pseudomonas* species have been reported to biosynthesize medium-chain-length PHAs containing various functional groups at the terminal position in the presence of carbon sources containing these functional groups (Kim et al. 2000; Lenz et al. 1992). In addition, PHAs with pendant functional groups have been obtained by chemical modification of PHA with unsaturated side chains (Hany et al. 2004; Park et al. 1998). Recently, dihydroxybutyrates (DHBAs) have been produced in *E. coli* expressing the *pct*, *phaB*, and *tesB* genes grown on glucose supplemented with glycolate (Martin et al. 2013). The chemical structure of DHBA is similar to 3HB, which is the most abundant component of PHA in nature, with an additional hydroxyl group. Therefore, modifying a pathway for the biosynthesis of PHA containing DHBA monomers may be useful for producing a novel PHA with pendant hydroxyl groups.

In this study, an artificial pathway for the generation of DHBA-CoA from glycolate was constructed by introducing *pct* from *M. elsdenii* and *bktB* from *R. eutropha* H16 into *E. coli* JM109. The *phaCAB* operon from *R. eutropha* H16 was also introduced to generate the PHA biosynthesis pathway. Then, PHA biosynthesis was conducted using glycolate as the sole carbon source. Based on the current results, the PHA content was relatively low (<1 wt%) compared with the samples using glucose as a carbon source (5.5 wt%). In PHA biosynthesis pathway, provision of hydroxyalkanoate monomers is supplied in the cells via metabolism of carbon sources. The type of carbon source used for cultivation effects on PHA production. Therefore, the low PHA content was likely due to the pathway for supplying PHA biosynthesis competes for glycolate metabolism (Hansen and Hayashi 1962). NMR analyses revealed that a small fraction of 2,3-DHBA (1 mol%) was incorporated into PHA. In a previous report, the recombinant strains has been found to produce both 3,4-DHBA and 2,3-DHBA in similar ratios (Martin et al. 2013). This report indicates that 3,4-DHBA-CoA is not a favorable substrate for PHA synthase compared with 2,3-DHBA. The low composition of DHBA was likely due to high metabolic flux of (*R*)-3HB-CoA, the most preferred substrate for PHA synthase, owing to the expression of the *R. eutropha phaAB1* genes. It has been reported that efficient PHA production can be achieved by enhancing metabolic flux to supply intermediates sufficient for PHA biosynthesis and by expressing PHA synthase that has high substrate specificity toward the desired monomer (Fukui et al. 1999; Park et al. 2012). We therefore developed strategies for supplying more DHBA monomers from glycolate and improving substrate specificity toward DHBA-CoA. It is possible to decrease the carbon flux from glycolate to acetyl-CoA by disrupting the glycolate oxidase gene, which catalyzes the first reaction, in which glycolate is converted to glyoxylate. Furthermore, the application of another PHA synthase may improve the incorporation of DHBA monomers into PHA. These strategies will be investigated in the future.

The number-average molecular weight (*M*_*n*_) of the polymer in the present study was lower than that synthesized by recombinant *E. coli* TOP10 using glucose (*M*_*n*_ = 2.5 × 10^6^ g/mol and PDI = 1.6) (Ushimaru et al. 2014). The decrease in molecular weight and broader molecular weight distribution may be due to interference with the polymerization reaction of PHA synthase by the incorporation of 3HV and 2,3-DHBA monomers into PHA. In addition, the unusual carbon source also resulted in a decreased polymer molecular weight.

The melting and glass transition temperatures of the polymer did not significantly differ from those of P(3HB), even with the low composition of the DHBA monomer units. Both of these thermal decomposition processes depended on the monomer composition. Our results indicated that the thermal decomposition temperature of P(3HB-*co*-1 mol% 3HV-*co*-1 mol%-2,3-DHBA) was higher than that of P(3HB) (Table 5); hence, incorporation of 2,3-DHBA affects the thermal degradation process of PHA. However, Lee et al. have reported that high contents of hydroxyl groups reduces the thermal stability of PHA (Lee et al. 2001). The hydroxyl groups are able to react with the ester linkages in the main chain, resulting in molecular weight loss and volatilization. Therefore, the appropriate DHBA composition in PHA should be adjusted for the practical application of this polymer.

In conclusion, this is the first report of the microbial production of P(3HB-*co*-3HV-*co*-2,3-DHBA) from glycolate. We constructed an artificial pathway that generated DHBA-CoA by introducing the *pct* gene from *M. elsdenii* and the *bktB* gene from *R. eutropha* H16 into *E. coli* JM109. The PHA production was approximately 1 wt% of the dry cell weight, and the DHBA composition was approximately 1 mol%. Further metabolic engineering to supply more DHBA monomers and improve PHA production will be developed in future studies. The thermal properties of PHAs containing DHBA were also characterized, thus providing valuable information for designing new types of PHAs. This study provides new insights into synthesizing PHA containing hydroxyl groups from renewable resources via designed metabolic pathways.
